# Onset and long-term duration of immunity provided by a single vaccination with recombinant a Marek’s disease virus with REV-LTR insertion

**DOI:** 10.3389/fvets.2024.1510834

**Published:** 2024-12-13

**Authors:** Jun Dai, Cuiping Song, Lei Tan, Yingjie Sun, Ning Tang, Yang Qu, Ying Liao, Xusheng Qiu, Chan Ding

**Affiliations:** ^1^Shanghai Veterinary Research Institute, Chinese Academy of Agricultural Sciences, Shanghai, China; ^2^Experimental Animal Center, Zunyi Medical University, Zunyi, China; ^3^Jiangsu Co-innovation Center for Prevention and Control of Important Animal Infectious Diseases and Zoonoses, Yangzhou University, Yangzhou, China

**Keywords:** Marek’s disease virus, REV-LTR, vaccine, onset of immunity, duration of immunity

## Abstract

Marek’s Disease (MD), caused by Marek’s disease virus (MDV), is a highly contagious lymphoproliferative disease in poultry. Despite the fact that MD has been effectively controlled by vaccines, the virulence of field isolates of MDV has continued to evolve, becoming more virulent under the immune pressure of vaccines. Our previous research has confirmed that the recombinant rMDV strain with REV-LTR insertion can be used as a live attenuated vaccine candidate. The aim of this research was to evaluate the onset and duration of immunity of the rMDV strain through two experiments. In both experiments, 1-day-old SPF chickens were vaccinated subcutaneously with the rMDV strain at a dose of 3,000 Plaque Formation Unit (PFU) per chick in 0.2 mL of the MD diluent. Then, in Experimental design 1, the chicks in the groups Vac-3d/CC-3d, Vac-5d/CC-5d, and Vac-7d/CC-7d were challenged separately with 500 PFU vvMDV strain MD5 at 3 days, 5 days, and 7 days after vaccination; in Experimental design 2, the chicks in group Vac-60d/CC-60d, Vac-120d/CC-120d, and Vac-180d/CC-180d were challenged at 60 days, 120 days, and 180 days after vaccination. The clinical symptoms and weight gain of chickens in each group were observed and recorded. The results showed that the rMDV strain with REV-LTR insertion provides protection starting from 3 days of age and achieves good immune effects at 5 days of age after 1-day-old immunization, and the immunization duration can reach for at least 180 days. Given age-related resistance, it can be confirmed that our vaccine can actually provide lifelong immunity. This study provides valuable insights into the onset and duration of immunity of the rMDV strain, which will provide a basis for the development and improvement of MD vaccines.

## Highlights

- First evaluate the duration of immunity of the rMDV strain in old chickens
- Vaccine rMDV with REV-LTR insertion can provide lifelong immunity
- Provide a basis for the development and improvement of MD vaccines

## Introduction

Marek’s disease (MD) is a highly contagious lymphoproliferative disease of chickens and is characterized by immunosuppression, neurological lesions, and rapid onset of T cell lymphoma in visceral organs and skin (1). Its causative agent, Marek’s disease virus (MDV), belongs to the *Mardivirus* genus of the *Alphaherpesvirinae* sub-family in the *Herpesviridae* family. Within the *Mardivirus* genus, there are three highly related species which correspond to previously described MDV serotypes: MDV serotype 1 (MDV-1, or Gallid herpesvirus 2, GaHV-2), MDV serotype 2 (MDV-2 or GaHV-3), and turkey herpesvirus (HVT, also known as Meleagrid herpesvirus 1, MeHV-1 or MDV-3) (2, 3). MDV-1 includes oncogenic viruses of variable virulence, which was further classified into mild (m), virulent (v), very virulent (vv), and very virulent plus (vv+) pathotypes (4). MDV-2 and HVT are non-oncogenic virus isolates from chickens and turkeys, respectively.

MD is the first ever viral oncogenic disease that was successfully controlled by vaccination. The CVI988/Rispens (an attenuated MDV-1 strain), SB-1 (an MDV-2 strain), and HVT strains are widely used for monovalent and combined MD vaccines and provide effective and lifelong immunity (5–8). These vaccines protected the immunized flocks against field virulent isolates with significant reduction of morbidity and mortality rates and effective inhibition of the formation of tumors. It was reported that the losses from MD infection in the United States sharply decreased from 1.5% in 1970 to 0.003% in 2006 after widespread administration of MD vaccines (9). However, MD continues to have a significant economic impact on the poultry industry worldwide due to the cost of vaccination and occasional outbreaks (1).

It is widely accepted that the MD vaccines do not provide sterilizing immunity: the virulent viruses could replicate in the vaccinated chickens and keep shedding, which may contribute to the evolution of viruses toward greater virulence (6, 10). In fact, the field strains of MDV-1 were continuously evolving with increased virulence in the last several decades (8, 11–14), even with the widespread use of effective MD vaccines. In the 1970s, HVT was first used as a successful vaccine to protect against vMDV isolates. A decade later, the HVT/MDV-2 bivalent vaccines had to be commercialized to deal with the emergence of vvMDV filed isolates. In the early 1990s, the CVI988/Rispens, a cell culture passage attenuated MDV-1 strain, was recommended to protect against the infection of vv + MD field strains (7). Up to now, CVI988/Rispens vaccine strain is still considered the gold standard of MD vaccines because of its superior protection. Nevertheless, the highly virulent MDV strains have been isolated in commercial chickens vaccinated with HVT plus CVI988 since 2017 (12–16). It is predicted that a more-virulent MD field strain that emerges in the future may lead to devastating consequences for the poultry industry, thus it is necessary to develop novel and more effective strategies against MDV.

Based on the long-term protective effect and safety of CVI988/Rispens strain (the gold standard MD vaccine), it may be one of the most effective ways to develop genetically engineered vaccines based on CVI988/Rispens vaccine strain to protect against emerging more-virulent isolates. Since the insertional mutagenesis of retroviral promotor/enhancer sequences in the MDV genome has been shown to enhance virus replication in the chickens (17–20), a recombinant CVI988/Rispens (rMDV) strain was constructed in our previous study, in the genome of which two copies of the long terminal repeat (LTR) from avian reticuloendotheliosis virus (REV) were inserted (21). The REV-LTR insertions increased the horizontal transmission of MD (22) and enhanced the virus replication of the rMDV strain probably due to a strong promoter and enhancer effect (17–21, 23), but had no obvious effect on virus virulence (21). The previous results showed that the rMDV was safe for vaccinated chickens and showed better protective effect compared to the parental CVI988/Rispens strain (21).

The aim of this study presented here was to evaluate the onset and duration of immunity of the rMDV strain. It is generally believed that herpes virus is lifelong immunity; however, since the molecular manipulation and modifications on the recombinant viruses may have changed their latency and reactivation (8, 21, 24), whether recombinant viruses can provide long lasting and highly protection efficacy has not been determined. This study of the duration of immunity for recombinant MD will provide a basis for the development and improvement of MD vaccines and therapies.

## Materials and methods

### Ethics statement

This study was carried out in strict accordance with recommendations from the Guide for the Care and Use of Laboratory Animals of Shanghai Veterinary Research Institute, the Chinese Academy of Agricultural Sciences (SHVRI, CAAS). The protocols were approved by the Institutional Animal Care and Use Committee (IACUC) of SHVRI, CAAS. Specific pathogen-free (SPF) chicken embryos for preparation of SPF chickens and CEF cells were from Beijing Boehringer Ingelheim Viton Biotechnology Co., Ltd. (Beijing, China). The SPF chickens were housed in isolators under controlled temperatures (28°C–30°C) with a 12 h light/dark cycle and were given free access to food and water during the study.

### Viruses and vaccines

The rMDV strain was preserved in our laboratory and reported previously (21), and the vvMDV MD5 strain was provided by Professor Cui of Shandong Agriculture University. This strain is oncogenic in unvaccinated chickens and can also induce atrophy of lymphoid organs (25, 26). Viral titers were determined by a viral plaque assay (27) and stored in liquid nitrogen until use.

### Experimental design 1

To determine the onset of immunity of the rMDV strain, 105 1-day-old SPF chicks were randomly divided into 7 groups equally (15 for each group) and raised in 7 isolators separately. When the chicks were 1-day-old, the chicks in Vac-3d, Vac-5d and Vac-7d groups were inoculated subcutaneously with rMDV strain in the back and neck at the dose of 3,000 PFU per chick in 0.2 mL of the MD diluent (Boehringer Ingelheim, China), whereas chicks in 4 control groups were inoculated with 0.2 mL MD diluent. At 3, 5 and 7 days after vaccination, the chicks in group Vac-3d/CC-3d, Vac-5d/CC-5d and Vac-7d/CC-7d were challenged separately via intra-abdominal injection with 500 PFU vvMDV MD5 to observe the protective effects of rMDV (Table 1). In addition, a group of unvaccinated and unchallenged chickens (Control-A) was raised and taken as a healthy control. All chickens were kept under the same conditions and were evaluated and recorded daily for symptoms of MD.

**Table 1:** Experimental design for the onset of immunity assay.

Group	Vaccine		Challenge		
	Strain	Dose/age	Strain	Dose/age	Route
Vac-3d	rMDV	3,000/1d	MD5	500/3d	Intra-abdominally
Vac-5d	rMDV	3,000/1d	MD5	500/5d	Intra-abdominally
Vac-7d	rMDV	3,000/1d	MD5	500/7d	Intra-abdominally
CC-3d	–	–	MD5	500/3d	Intra-abdominally
CC-5d	–	–	MD5	500/5d	Intra-abdominally
CC-7d	–	–	MD5	500/7d	Intra-abdominally
Control-A	–	–	–	–	–

(1) chicks in 1-day-old were inoculated subcutaneously with rMDV strain, the chicks in group Vac-3d (At 3 days after vaccination), Vac-5d (At 5 days after vaccination) and Vac-7d (At 7 days after vaccination) were challenged vvMDV MD5 to observe the protective effects of rMDV. (2) chicks in 1-day-old were inoculated with 0.2 mL MD diluent, the chicks in group CC-3d (3-days-old), CC-5d (5-days-old) and CC-7d (7-days-old) were challenged vvMDV MD5 to observe the protective effects of rMDV. (3) Control-A: a group of unvaccinated and unchallenged chickens was raised and taken as a healthy control.

The body weight of the chickens in the different groups were measured separately at 14, 28, 42 and 60 days post last challenge (namely 21, 35, 49 and 67 days old) to evaluate the protective effect of the rMDV strain on growth rate. At 60 days post-challenge, all the surviving chickens were sacrificed for necropsy. The presence of gross lesions was evaluated, and then all bursa and spleen from each chicken were collected and weighed. The relative weights of the bursa and spleen to the whole body were further determined. At last, cumulative mortality and gross tumor rate were used for comparing the protective effect of rMDV against the attack of MD5 at different days after vaccination.

### Experimental design 2

To determine the duration of immunity of the rMDV strain, 115 1-day-old SPF chicks were randomly divided into 7 groups (20 for each group of Vac-60d/CC-60d, 15 for each of Vac-120d/CC-120d and Vac-180d/CC-180d) and raised in 7 isolators separately. The 1-day-old chicks in Vac-60d, Vac-120d and Vac-180d groups were inoculated subcutaneously with rMDV strain in the back and neck at the dose of 3,000 PFU per chick in 0.2 mL of the MD diluent (Boehringer Ingelheim, China), whereas chicks in CC-60d, CC-120d, CC-180d and Control-B groups were inoculated with 0.2 mL MD diluent. At 60, 120 and 180 days after vaccination, the chicks in group Vac-60d/CC-60d, Vac-120d/CC-120d and Vac-180d/CC-180d were challenged intra-abdominally with 500 PFU vvMDV MD5 to observe the protective effects of rMDV (Table 2). All chickens were kept under the same conditions and were evaluated and recorded daily for symptoms of MD. At 60 dpi, all the surviving chickens of each challenged group as well as 5 non-challenged chickens in group Control-B were sacrificed for necropsy after being weighed. The presence of gross lesions was evaluated, and the cumulative mortality and gross tumor rate were used for comparing the protective effect of rMDV against the attack of MD5 at different days after vaccination.

**Table 2:** Experimental design for the duration of immunity assay.

Group	Vaccine		Challenge			
	Strain	Dose/age	Strain	Dose/age	Route	Culling of age (dpi)
Vac-60d	rMDV	3,000/1d	MD5	500/60d	Intra-abdominally	120
Vac-120d	rMDV	3,000/1d	MD5	500/120d	Intra-abdominally	180
Vac-180d	rMDV	3,000/1d	MD5	500/180d	Intra-abdominally	240
CC-60d	–	–	MD5	500/60d	Intra-abdominally	120
CC-120d	–	–	MD5	500/120d	Intra-abdominally	180
CC-180d	–	–	MD5	500/180d	Intra-abdominally	240
Control-B	–	–	–	–	–	120, 180 and 240

### Evaluation standard

The non-challenged Group Control-A and Control-B were kept in the isolators under the same conditions with challenged group, and taken as healthy controls. All chickens were monitored daily for symptoms of MD: chickens with ocular lesions, and those that had no observable lesions but presented pathological changes during the experiment were deemed positive; chickens that had no clinical observations, no gross lesions, and no pathological changes were considered negative. The suspected samples were subject to confirmation by laboratory tests.

MD incidence criteria was as follows: experimental chicken death (excluding non-specific death), severe weight loss, paralysis syndrome, thymus and bursa Fabricius atrophy, diffuse enlargement of internal organs (including liver, kidneys, heart, spleen, ovaries, etc.) or tumors, peripheral nerve stripes, and swelling signified the onset of MD.

### Statistical analysis

The morbidity, mortality, mean body and organ weights for each group were compared using two-way ANOVA and Turkey’s multiple comparisons tests. All graphs and statistical analyses were generated with Prism 7 (GraphPad Software).

## Results

### The onset of immunity of the rMDV strain

#### Effects of the rMDV vaccination against early challenge on growth and weight gain

**Figure 1:**
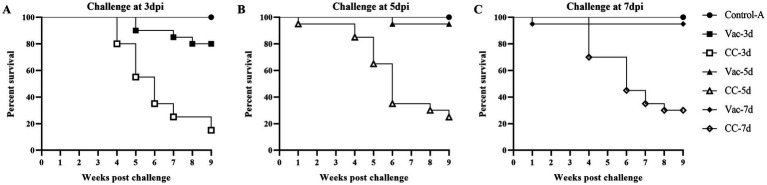
Weight gain of experimental SPF chickens. The chicks in Vac-3d, Vac-5d and Vac-7d groups were inoculated subcutaneously with rMDV strain in the back and neck at the dose of 3,000 PFU at 1 day of age, whereas chicks in CC-3d-4 were inoculated MD diluent. At 3, 5 and 7 days after vaccination, the chicks in group Vac-3d/CC-3d **(A)**, Vac-5d/CC-5d **(B)** and Vac-7d/CC-7d **(C)** were challenged separately via intra-abdominal injection with 500 PFU MD5. The body weight of the chickens in the different groups were measured separately at 14, 28, 42 and 60 days post last challenge (namely 21, 35, 49 and 67 days old). Results represent mean value with error bars representing standard error of the mean. The mean body weights for each group were compared using two-way ANOVA and the significant differences were marked on the top of the columns. Significant differences are indicated by “*” (*p* < 0.05), “**” (*p* < 0.01), “****” (*p* < 0.0001).

In order to determine the onset of protection provided by the rMDV strain, 1-day-old SPF chickens were vaccinated subcutaneously and then challenged with 500 PFU vvMDV strain MD5 at 3d (Vac-3d/CC-3d), 5d (Vac-5d/CC-5d) and 7d (Vac-7d/CC-7d) after vaccination. The clinical symptoms and weight gain of chickens in each group were observed and recorded. Since the 2nd week after last challenge, clinic symptoms were gradually observed in all the chickens in the unvaccinated control groups (CC-3d/CC-7d), such as listlessness, depression, messy feathers, incoordination or stilted gait, emaciation, paralysis and death, which lasted 6 weeks. In the vaccination groups, there were 4 chicks in Vac-3d and 1 chick in Vac-5d that showed obvious clinical symptoms since the 3^rd^ week after challenge, and all the other vaccinated chickens appeared as healthy as the unchallenged control ones. The challenged and unchallenged chickens were weighed at 14, 28, 42 and 60 days post last challenge (namely 21, 35, 49 and 67 days old), which were then used for body weight gain chart and statistical analysis, as shown in Figure 1. The results showed that the body weights of chickens in three challenge control groups (CC-3d-CC-7d) were lower than that of the vaccinated chickens (Vac-3d-Vac-7d) and unchallenged chickens (Control-A) since about 3rd week after challenge, and the differences started to be significant in the 4th week (*p* < 0.01). The body weight of chickens in 3d-vaccinated (Vac-3d) and 5d-vaccinated (Vac-5d) chickens were significantly lower than that of control chickens at 42 days post the last challenge; however, the body weight gain recovered when the chickens were 60 days post last challenge. Apart from these, there was no significant differences on body weight gain between all the other vaccinated chickens and unchallenged control ones after challenge.

#### Effects of the rMDV vaccination against early challenge on mortality

**Figure 2:**
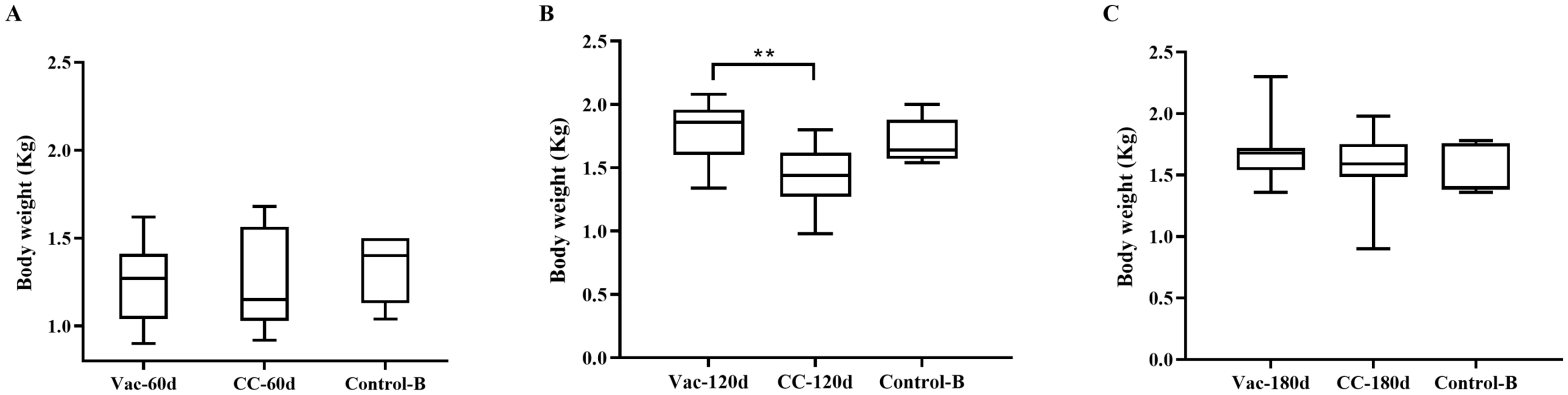
Survival curves of rMDV-vaccinated and unvaccinated SFP chickens after challenge of vvMDV MD5 strain. At 3 days **(A)**, 5 days **(B)** and 7 days **(C)** post inoculation (dpi), the chicks in group Vac-3d/CC-3d, Vac-5d/CC-5d and Vac-7d/CC-7d were challenged separately via intra-abdominal injection with 500 PFU MD5, and then observed for 60 days. A group of unvaccinated and unchallenged chickens (Control-A) were kept under the same conditions and taken as a healthy control.

Figure 2 provides a summary of the mortality rate in the experimental chickens within each group. At 1 week after challenge, 1 chick was found dead in CC-5d group but no pathological changes were observed during the necropsy. In the CC-3d-CC-7d group, deaths began to occur during the fourth week after challenge. Over a period of 60 days following the challenge, a total of 17, 15 and 14 chickens died in the CC-3d-CC-7d groups, respectively. In the Vac-3d group, a total of 4 vaccinated chickens were dead since the 3rd week after challenge at 3 days of age; while one chicken died in the Vac-5d group at the sixth week after challenge at 5 days of age. The majority of chicken death was observed within 4 to 6 weeks post-challenge, specifically, during the tumor stage of MD. The dead chickens showed grossly enlarged spleens with lymphoid hyperplasia, atrophy of the bursa of Fabricius and diffuse tumor lesions in the hearts, livers, and kidneys at necropsy.

**Figure 3:**
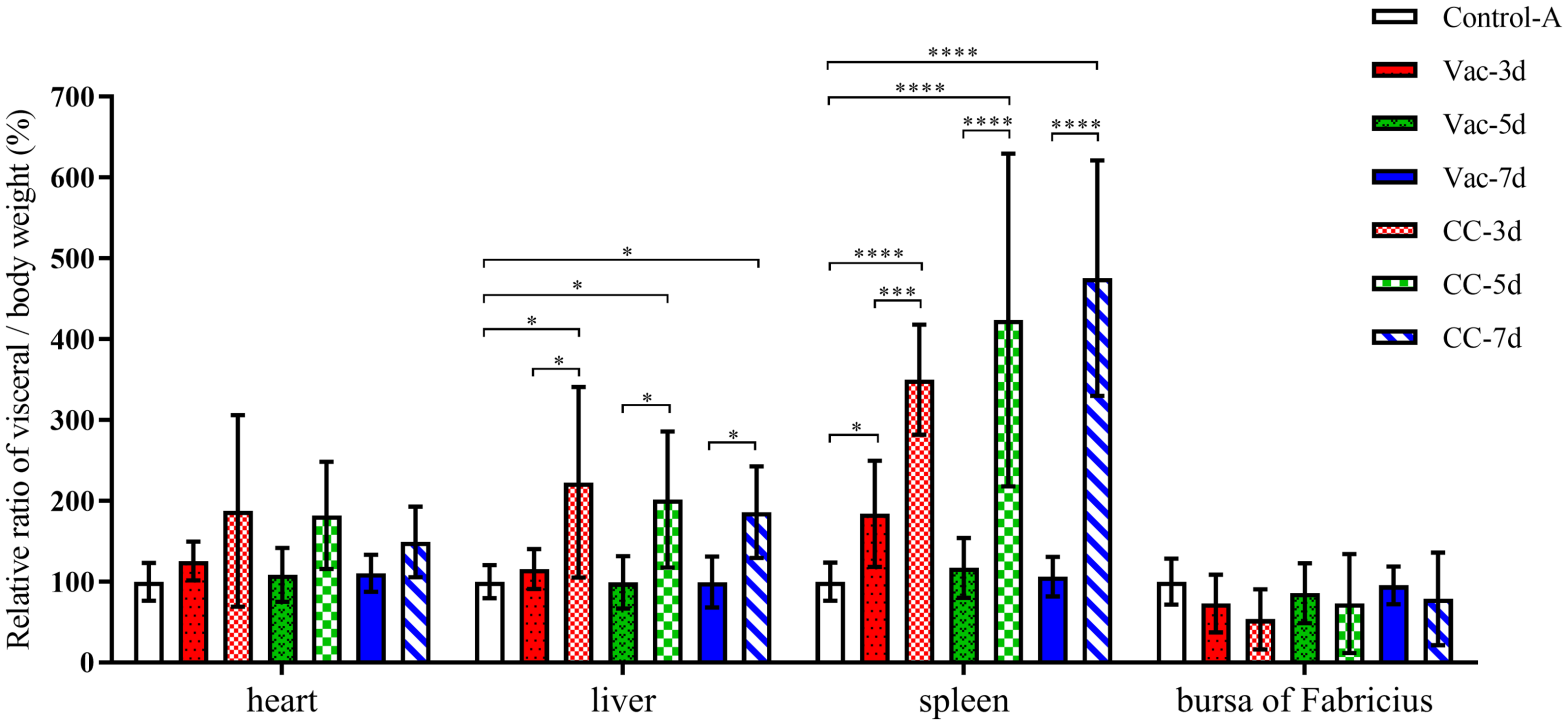
Relative ratio of organ weights compared to body weights. At 60 days post-challenge, all the surviving chickens in Vac-3d/CC-3d, Vac-5d/CC-5d, Vac-7d/CC-7d and healthy control Control-A were sacrificed for necropsy after weighing. The hearts, livers, spleens and bursa were collected and weighed. The relative ratio of organ to body weight was determined as follows: $\text{Relative}\;\text{ratio}=\frac{\text{organ}/\text{body weight of each chicken}}{\text{the mean value of organ}/\text{body weight of the control group}}\times 100\%$. Results represent mean value with error bars representing standard error of the mean. The values for each group were compared using two-way ANOVA and the significant differences were marked on the top of the columns. Significant differences are indicated by “*” (*p* < 0.05), “***” (*p* < 0.001), **** (*p* < 0.0001).

All surviving chickens were sacrificed for necropsy to evaluate the presence of gross lesions after 60 days of observation. There were only 3, 5 and 6 unvaccinated chickens in CC-3d-CC-7d survived from MD5 attack, which showed obvious clinic symptoms of listlessness, emaciation or paralysis. In those chickens, visceral tumors were found in 2, 3 and 2 surviving chickens in the CC-3d-CC-7d group, respectively. As shown in Figure 3, the weights of livers and spleens significantly increased in CC-3d, CC-5d and CC-7d groups, and the bursa weights in which were a little heavier than that of Vac-3d, Vac-5d, Vac-7d and Control-A. In the vaccinated chickens, only one chicken in each group of Vac-3d-Vac-7d was found visceral tumors; nevertheless, no significant differences were observed in the weights of their livers, spleens and bursa of Fabricius when compared to the unchallenged group. In addition, although some of the birds had paralysis of the neck and weakness of the legs, but no obvious sign of neurological lesions was observed in all these dead chickens. Cumulative mortality and morbidity in each group were summarized in Table 3, and the immune protection index of each immune group was calculated for comparing the protective effect of rMDV stain against the attack of vvMDV strain MD5 at different days of age.

**Table 3:** Protective efficacy of rMDV strain against vvMDV MD5 strain in SPF chickens at 3, 5, and 7 days after vaccination.

Group	Vaccination	Challenge	Morbidity and Morality			PI (%)^3^
			Tumors^1^	Death	Incidence (%)^2^	
Vac-3d	rMDV	MD5	1	4	5/20 (25)	63.2
CC-3d	–	MD5	2	17	19/20 (95)	–
Vac-5d	rMDV	MD5	1	1	2/20 (10)	88.9
CC-5d	–	MD5	3	15	18/20 (90)	–
Vac-7d	rMDV	MD5	1	0	1/20 (5)	93.75
CC-7d	–	MD5	2	14	16/20 (80)	–
Control-A	–	–	0	0	0/15	–

^1^Tumors = Incidence of MD specific gross tumors in the survived chickens; ^2^Incidence (%) = Percentage of the sick or dead chickens until 60 days post-challenge; ^3^PI = Protective index. $\text{PI}\left(\%\right)=\frac{\text{Incidence of challenge control}-\text{Incidence of each experimental group}}{\text{Incidence of challenge control}}\times 100\%$.

### The duration of immunity of the rMDV strain

#### The pathogenicity of MD5 in old chickens

**Figure 4:**
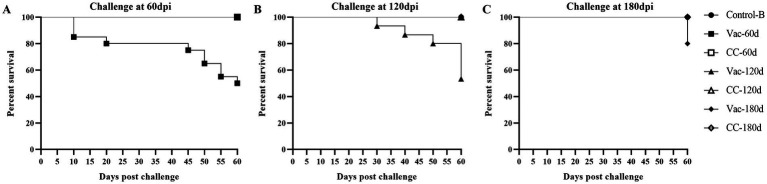
Survival curves of rMDV-vaccinated and unvaccinated old SFP chickens after challenge of vvMDV MD5 strain. At 60 days **(A)**, 120 days **(B)** and 180 days **(C)** post inoculation (dpi), the chicks in group Vac-60d/CC-60d, Vac-120d/CC-120d and Vac-180d/CC-180d were challenged separately via intra-abdominal injection with 500 PFU MD5, and then observed for 60 days. A group of unvaccinated and unchallenged chickens (Control-B) were kept under the same conditions and taken as a healthy control.

In experiment 2, in which unvaccinated SPF chickens of 60-days (CC-60d), 120-days (CC-120d) and 180-days (CC-180d) old were challenged by intra-abdominal inoculation, mortality caused by MD infection was observed in all these three challenge control groups, although age-related resistance was apparent. Since the 10d and 28d post-challenge, the clinic symptoms were gradually observed in the CC-60d and CC-120d group, such as listlessness, messy feathers, and incoordination or stilted gait and death (Figure 4). The dead chickens showed grossly enlarged spleens and diffuse tumor lesions in the hearts, livers, and kidneys at necropsy. At 60 days post-challenge, the surviving chickens were sacrificed for necropsy (Supplementary Figure S1). In the unvaccinated chickens that challenged at 60 days of age (CC-60d), the grossly enlarged spleens with diffuse tumors were found in 6 chickens. In the group challenged at 120 days of age, one chickens showed enlarged spleen and liver, and another one was extremely emaciated.

In the CC-180d group, in which the unvaccinated chickens were challenged at 180 days of age, all the old chickens appeared healthy throughout the experiment, but 3 chickens died suddenly at 60 days post-challenge. Furthermore, diffuse tumors were found in the hearts and spleens of all the 3 chickens during the necropsy at 60 days post-challenge.

The cumulative incidence and morality of MD in 60-, 120- and 180-days-old chicks were 80%/50, 53.3%/46.7 and 40%/20% (Table 4); in contrast, that of MD in 3-, 5-, and 7-days-old chicks were 95%/85, 90%/75 and 80%/70% in experiment 1 (Table 3).

**Table 4:** Protective efficacy of rMDV strain against vvMDV MD5 strain in old SPF chickens at 60, 120, and 180 days after vaccination.

Group	Vaccination	Challenge	Morbidity and morality			PI (%)^3^
			Tumors^1^	Death	Incidence (%)^2^	
Vac-60d	rMDV	MD5	0	0	0/20 (0)	100
CC-60d	–	MD5	6	10	16/20 (80)	–
Vac-120d	rMDV	MD5	0	0	0/15 (0)	100
CC-120d	–	MD5	2	7	9/15 (60)	–
Vac-180d	rMDV	MD5	0	0	0/15 (0)	100
CC-180d	–	MD5	4	3	7/15 (46.6)	–
Control-B	–	–	0	0	0/15	–

^1^Tumors = Incidence of MD specific gross tumors in the survived chickens; ^2^Incidence (%) = Percentage of the sick or dead chickens until 60 days post-challenge; ^3^PI = Protective index.

#### The protective effects of rMDV on vaccinated old chickens

**Figure 5:**
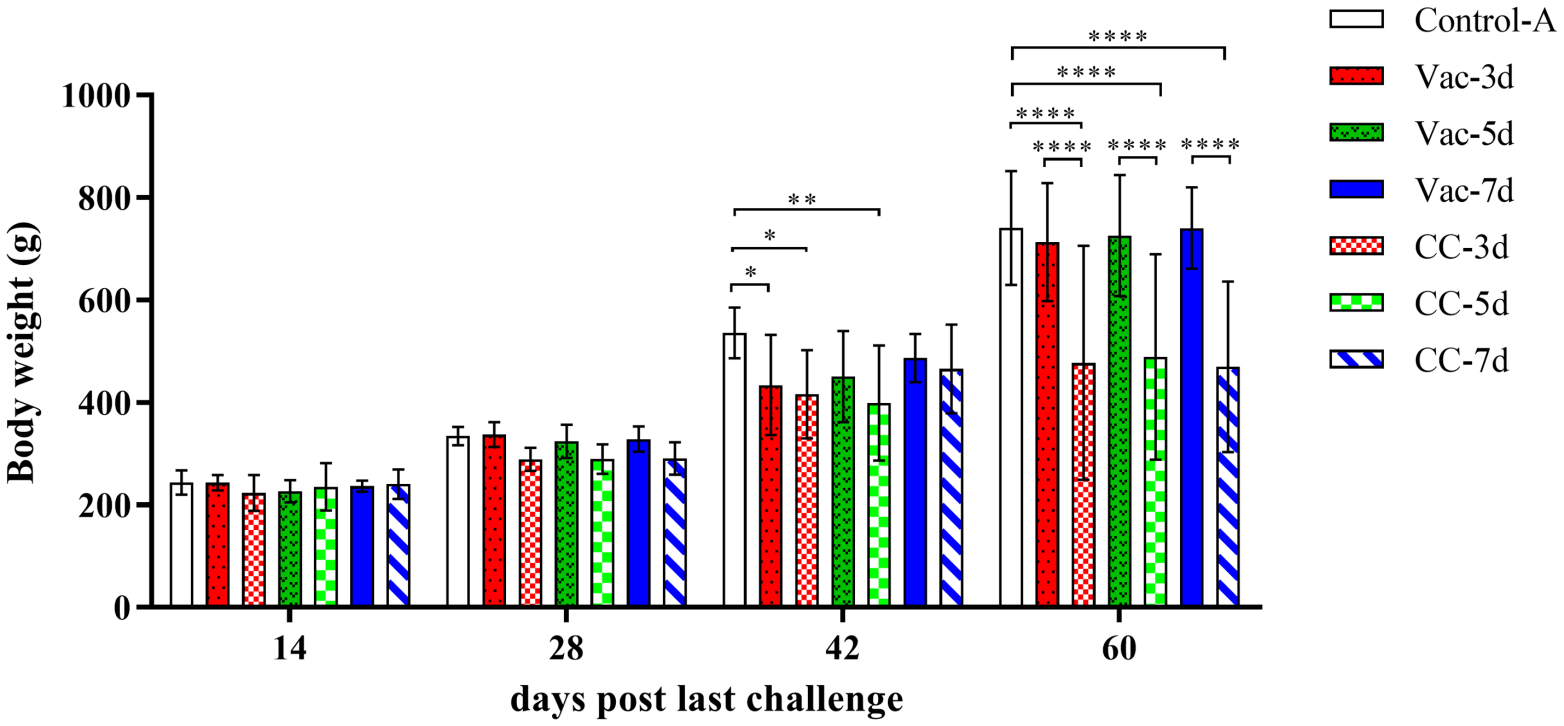
Weight of chicken carcass of each group at 120, 180 and 240 days of immunization duration. Results represent mean value with error bars representing standard error of the mean. The values for each group were compared using two-way ANOVA and the significant differences were marked on the top of the columns. Significant differences are indicated by “***” (*p* < 0.01).

All the vaccinated chickens in Vac-60d, Vac-120d and Vac-180d were as healthy as the unchallenged controls, and no chicken died during the 60-day observation period. The results of weighing at 60 days after challenge showed that there were no significant differences in body weight between Vac-60d, CC-60d and Control-B groups at 120 days of age, between Vac-120d and Control-B at 180 days of age, and between Vac-180d, CC-180d and Control-B at 240 days of age (Figure 5). The weight of chickens in CC-120d group was significantly lower than that of Vac-120d and Control-B groups. Furthermore, no any pathological lesion was found in the vaccinated chickens in Vac-60d, Vac-120d and Vac-180d at 60 days after challenge. Cumulative mortality and morbidity in each group were summarized in Table 4, and the immune protection index of each immune group was calculated for comparing the protective effect of rMDV stain for old chickens against the attack of vvMDV strain MD5. In addition, besides these confirmed cases, there was one visibly emaciated experimental chicken in each of the CC-120d and CC-180d groups, but no organ tumors were observed after dissection.

## Discussion

The MDV vaccine immunization does not provide sterilizing immunity; therefore, it is widely believed that the administration of vaccines accelerates the continuous evolution of virulence in circulating wild strains. In recent years, there has been a marked increase in the prevalence of MDV in China. Despite extensive vaccination campaigns aimed at reducing the impact of MD, numerous cases have arisen where wild strains of MDV have managed to evade the immune protection conferred by these vaccines (28). Consequently, the development of MDV vaccines is ongoing in response to new virulent circulating strains. Traditional technology is approaching its limits, and genetic modification has become the primary method for research and development in this field. There are numerous virulence gene knock-out or modified vaccines available, including artificially attenuated MDV-1 strains that have been widely utilized (8, 21, 24).

Evidence suggests that the incorporation of REV LTR sequences into MDV can enhance its proliferation efficiency and immune protective effect (17, 18, 23, 29–31). However, the precise mechanism underlying this synergistic replication is not yet fully understood. It is known that insertional mutagenesis of retroviral LTR sequences in the MDV genome may elevate virus replication in some unknown manner. This enhancement is not limited to recombinant viruses. In fact, it was also observed that co-infection of MDV and REV has been demonstrated to increase viral replication in co-infected cells (23). To date, there have been a number of studies trying to uncover the mechanism. Faiz et al. (32) evaluated the load of oncogenic MDV DNA in five commercial flocks that were vaccinated with CVI988 with or without REV-LTR insertion. They found that CVI988 with REV-LTR insertion tended to replicate in the thymus earlier than in other lymphoid organs. Previous research has indicated that the extensive replication in the lymphoid organs was associated with vaccine protection (33). The early and strong replication in the thymus may contribute to decrease oncogenic MDV load and might induce early immune responses (32). Furthermore, Su et al. (22) found that the REV-LTR insertions increased the horizontal transmission of MD; and however, the mechanism remain unclear.

Despite the enhancement of certain biological properties of MDV by the REV-LTR insertion, it does not elevate the virus virulence. In our previous studies, no significant virulence changes were found in the recombinant virus after the insertion of REV-LTR insertion (21). Moreover, the virulent MDV strains were partially attenuated after REV-LTR insertion. For example, the RM1 strain of MDV, a recombinant MDV obtained via co-cultivation of the JM/102 W strain of MDV with REV, was highly attenuated for oncogenicity but induced severe bursal and thymic atrophy (34). Further research is needed to understand the mechanism by which REV-LTR enhances the immune effect to develop better MDV vaccines.

Our previous studies have demonstrated that the safety and improvement of immune protection about the insertion of REV LTR into CVI988 (21). Beyond safety, another consideration when using recombinant MDV strains is the unknown impact of genetic recombination on the onset and persistence of immunity. In this study, we investigated the onset of protection and the duration of immunity of the rMDV strain, which was constructed from the vaccine strain CVI988/Rispens with REV-LTR inserted into its genome (21). This is the first study to describe the duration of immunity of the LTR-inserted recombinant MDV strain in older SPF chickens after vaccination at 1 day of age.

In experimental design 1, we initially evaluated the onset of immunity of the rMDV strain in chickens. One-day-old SPF chickens were immunized subcutaneously, and 500 PFU of virulent MDV Md5 was injected intraperitoneally on days 3, 5, and 7 after immunization. Clinical symptoms, weight gain, incidence, mortality, and autopsy were observed in each group of chickens to comprehensively assess the protective effect of rMDV against Md5 challenge. In the two groups challenged on the third day, the morbidity and mortality rates in the non-immunized group (CC-3d) reached 95 and 85%, respectively, while those in the immunized group (Vac-3d) were only 25 and 20%. In terms of the protection rate, immunization with the rMDV strain for 5 days can provide 93.75% protection against the virus, while immunization for 3 days can provide a protection rate of 63.2%. Therefore, our vaccine can take effect at 3 days of age and achieve good immune effects at 5 days of age. Early research indicates that MDV only generates stable protective effects on the 7th day after immunization (35). This may be due to the proliferative properties of MDV, as inoculated MDV viruses are active in the blood and take 7 days to colonize the T cells (6, 36, 37). It is supposed that the more rapid onset of protection induced by the rMDV5 strain might result from its proliferation efficiency.

The immune duration of MDV is generally considered to be lifelong (38, 39). However, due to age-related persistence, the actual immunoprotective efficiency in old age is difficult to determine (40). In experimental design 2, protective efficacy of older SPF chickens immunized with rMDV to the challenge of vvMDV MD5 strain. Surprisingly, the vvMDV MD5 strain has strong pathogenicity in older chickens, although chickens aged 60 days or older exhibited a delayed onset of the disease. It was also found that the incidences of MD in 120-, 180-, and 240-day-old non-immunized SFP chickens decreased progressively after being challenged with the vvMDV strain MD5. Regrettably, 60 days of observation is still insufficient, as the group challenged at 180 days old clearly did not enter the peak period of death.

Moreover, no MDV vaccines can provide sterilizing immunity, so it is unclear whether residual vv + MDV in large chickens will also cause cancer or pathological lesions. The results of the present study are consistent with previous work, where older chickens showed age-related resistance to MDV infection (39–41). However, the unexpectedly high incidence of MD observed in unvaccinated SPF chickens could potentially be attributed to the enhanced virulence of the vvMD strain, and/or the vulnerability and genetic susceptibility to MD of the SPF used in the experiment. Further work involving other genetic stocks of chickens is needed to confirm this observation.

In CC-120d and CC-180d group, chickens exposed at 120 days and 180 days of age had a lower incidence of mortality and gross lymphomas than did early exposed SPF chickens. The decreased susceptibility of older chickens corresponded with their age. It was reported that age resistance may well developed at 4 weeks of age in chickens of certain lines (42). The control chickens were healthy and had no gross tumors at necropsy, hence the observed resistance of older SPF chickens must not be attributed to the inhibitory effects of pre-existing antibody (43). It has been postulated that the time of exposure to MD infection influence the eventual outcome of disease, and certain age-related factors inhibits or delays the development of clinical disease (40, 42). The age-related resistance to MD infection was firstly reported by Sevoian and Chamberlain (44) and subsequently by Biggs and Payne (43). It was then firmly established by experiments that excluded prior infection of experimental chickens (41, 42, 45). Since older chickens were susceptible to infection with MDV and had microscopic lesions at termination, it is postulated that their resistance may have been based on lesion regression. In fact, previous studies have demonstrated that age-related resistance to MD was expressed through lesion regression (40, 46). It is noted that in a certain proportion of infected chickens, lymph-proliferative lesions in nerves and viscera may regress. However, the factors responsible for recovery from natural or experimentally induced MD have not been investigated.

The pathogenic effect of MD5 on chickens of different ages can be blocked by our vaccine immunization. In this study, one-day-old SPF chickens were immunized via subcutaneous injection and challenged with vvMDV Md5 at 60d, 120d, and 180d post-immunization. The clinical observation findings indicated that there was no discernible difference between the challenge group Vac-60d, −120d and -180d and the control group B, and no clinical manifestations of MD were identified. The results of body weight demonstrated that the body weight of the challenge group following immunization did not decline in comparison to the control group. The vaccine offers effective protection. According to the established criteria, more than 80% of the immune protection provided by the vaccine was determined to be effective, meaning that the rMDV with REV-LTR insertion provided effective protection for at least 180 days. Considering age-related resistance, we can confidently assert that this vaccine effectively provides lifelong immunity.

### Limitations of the study

For this study, we employed SPF chickens as the subjects of the experiment, which may differ from commercial breeds in terms of genetic background, vaccine response, and disease resistance. As a result, the practical efficacy of the vaccine in commercial farming environments would be quite different. The vaccine efficacy between SPF chickens and commercial strains will prove to be a promising topic for research in the future.

## Data Availability

The original contributions presented in the study are included in the article/Supplementary material, further inquiries can be directed to the corresponding authors.

## Supplementary material

SUPPLEMENTARY FIGURE S1 Pathological lesions observed in SPF chicken challenged with the MDV vvMD5 strain.
