# Supplementary material for: Onset and long-term duration of immunity provided by a single vaccination with recombinant a Marek’s disease virus with REV-LTR insertion
Source: Front Vet Sci. 2024 Dec 13;11:1510834. doi: 10.3389/fvets.2024.1510834 (PMC11681624; doi:10.3389/fvets.2024.1510834)
Supplement: Supplementary file 1 [file Presentation_1.pptx]

## Slide 1
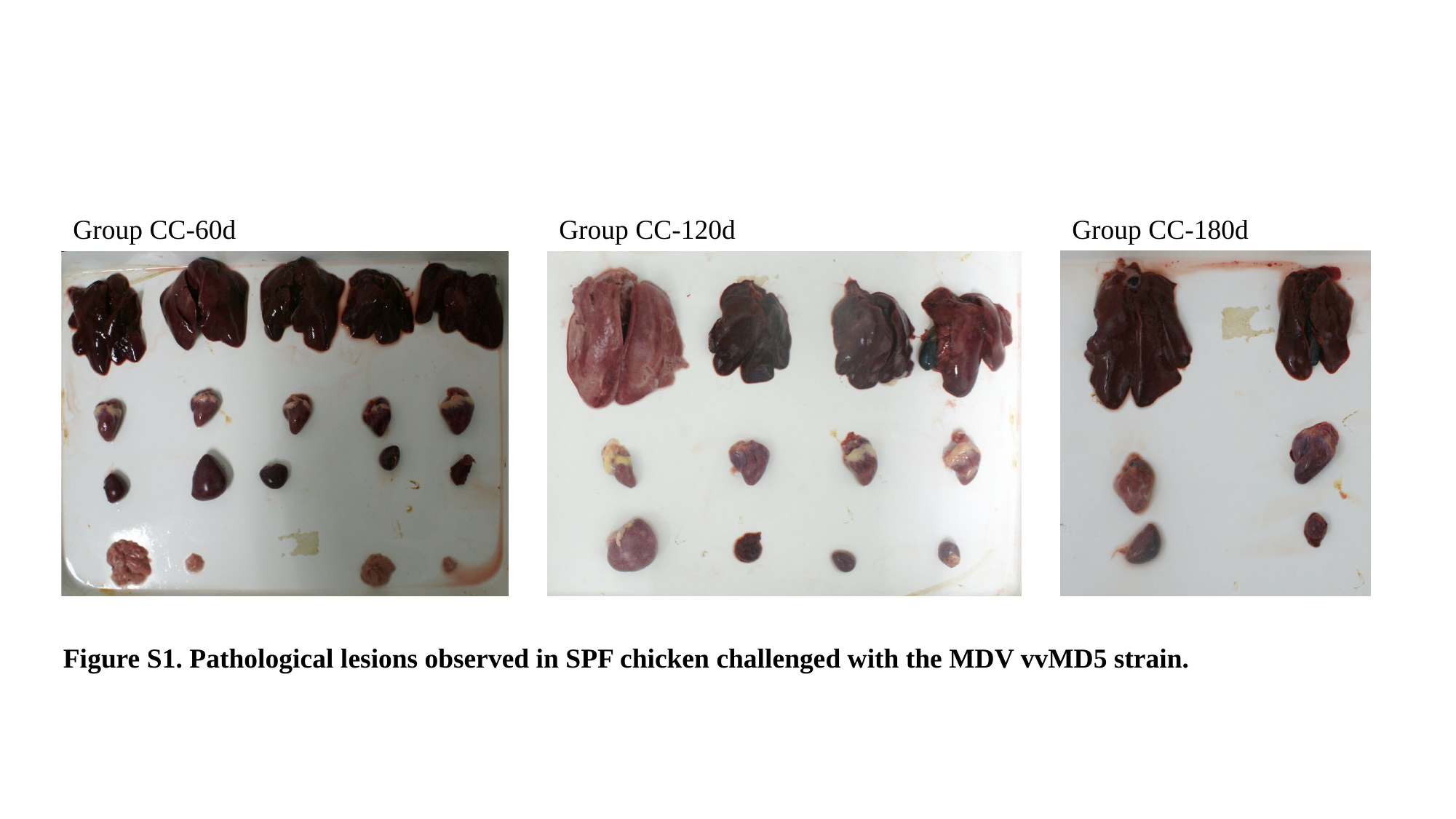

Group CC-120d
Group CC-180d
Group CC-60d
Figure S1. Pathological lesions observed in SPF chicken challenged with the MDV vvMD5 strain.
